# Open Pyelolithotomy in the Presence of a Retro-Renal Colon: A Safe Alternative in Resource-Limited Settings

**DOI:** 10.7759/cureus.15827

**Published:** 2021-06-22

**Authors:** Adrian R Rhudd

**Affiliations:** 1 Urology, Mount St John’s Medical Center, St John‘s, ATG

**Keywords:** retro-renal, colon, pyelolithotomy, percutaneous, stone, nephrolithotomy

## Abstract

A retro-renal colon (RRC) is a rare but important anatomical variant to consider when planning percutaneous stone surgery. CT scanning of the abdomen is critical to detect this and to plan the surgical approach to avoid injury to the colon. In this case report, a 38-year-old woman with large obstructing bilateral symptomatic renal stones was found to have a left retro-renal colon on image review prior to left percutaneous nephrolithotomy. An open pyelolithotomy was performed to surgically remove the stone and to prevent any injury to the colon. An RRC can occur in up to 16% of patients undergoing percutaneous renal access. A CT scan is important to rule out this anatomical variant and to allow for surgical planning to avoid injury to the colon. The modern era of endourology has brought a significant reduction in open stone surgery. An open pyelolithotomy is still a safe option for stone removal if an RRC is detected preoperatively. Injury to the colon during percutaneous access can most commonly be managed conservatively. Occasionally open intervention may be required. An RRC detected on a preoperative CT scan may influence surgical planning and an open pyelolithotomy can be performed to safely remove renal stones and prevent colonic injury, especially in resource-limited settings.

## Introduction

The surgical removal of large renal stones is often done via percutaneous nephrolithotomy (PCNL). Very rarely there are contraindications to performing a PCNL. Currently, open stone surgery is rarely performed with the availability of endourology expertise and resources. It may however find use in select circumstances, particularly in resource-limited settings. A retro-renal colon (RRC) is a rare anatomical variant that may make percutaneous access difficult or even complicated.

In this case report, a patient with a renal pelvic stone was noted on pre-operative CT imaging to have an RRC and the decision was made to perform an open pyelolithotomy, instead of a PCNL, to avoid injuring the colon and the potential complications associated with it. The literature involving the management of large renal stones in the presence of an RRC is reviewed.

## Case presentation

**Figure 1:**
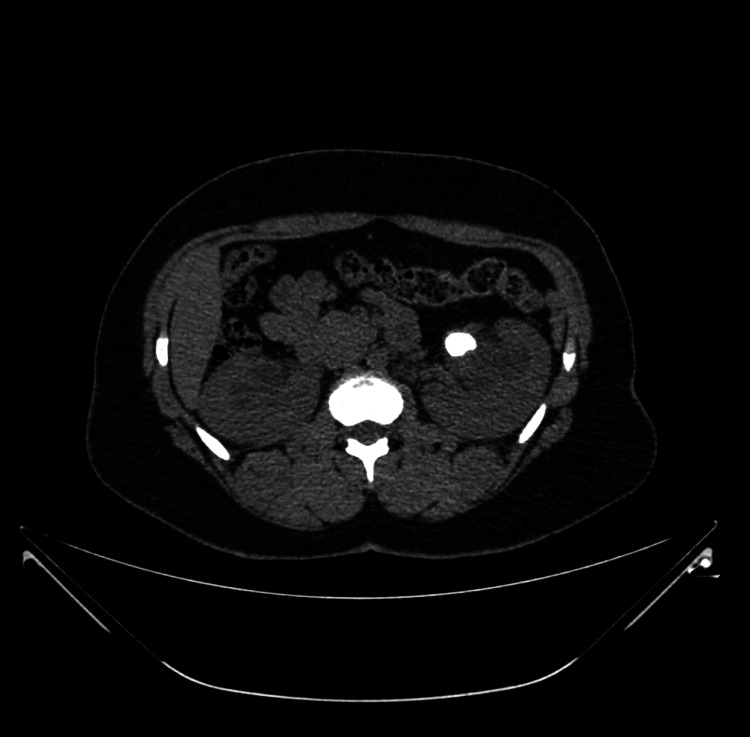
CT scan of abdomen - axial slice showing left renal pelvic stone with hydronephrosis

**Figure 2:**
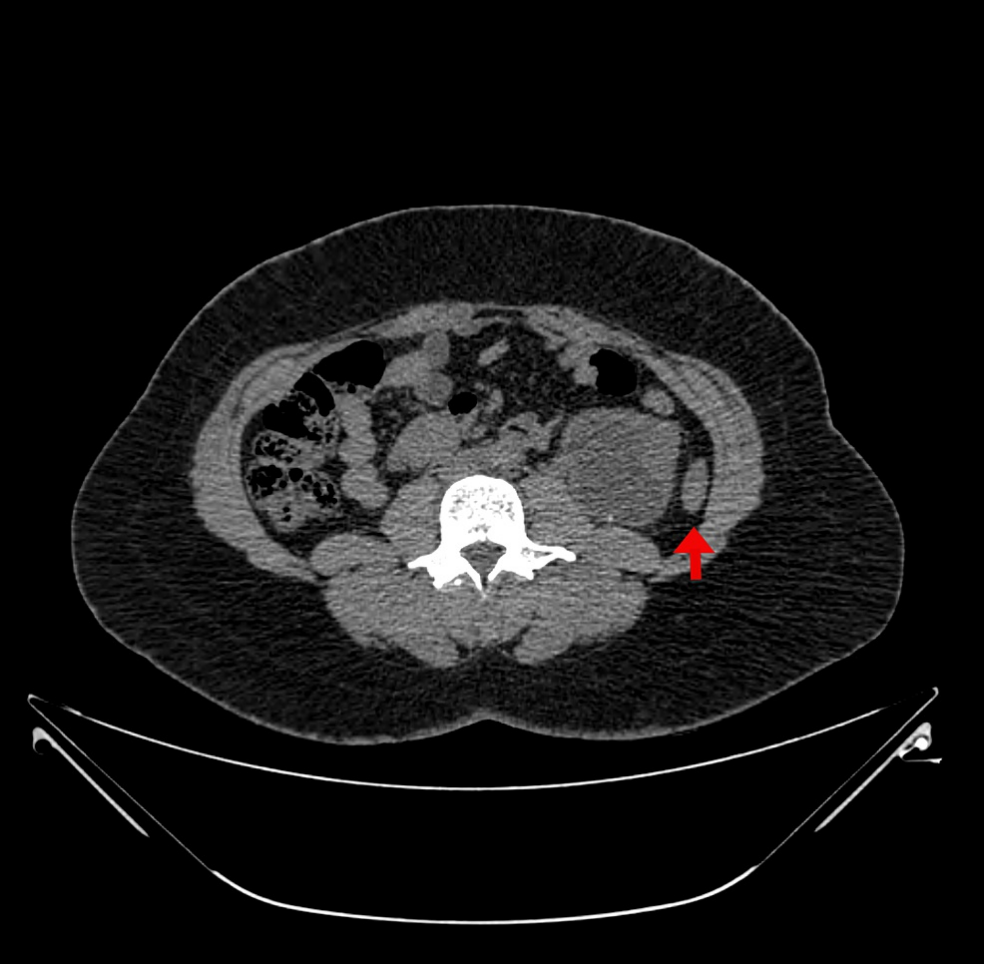
CT scan of abdomen - axial slice at lower pole showing left retro-renal colon

**Figure 3:**
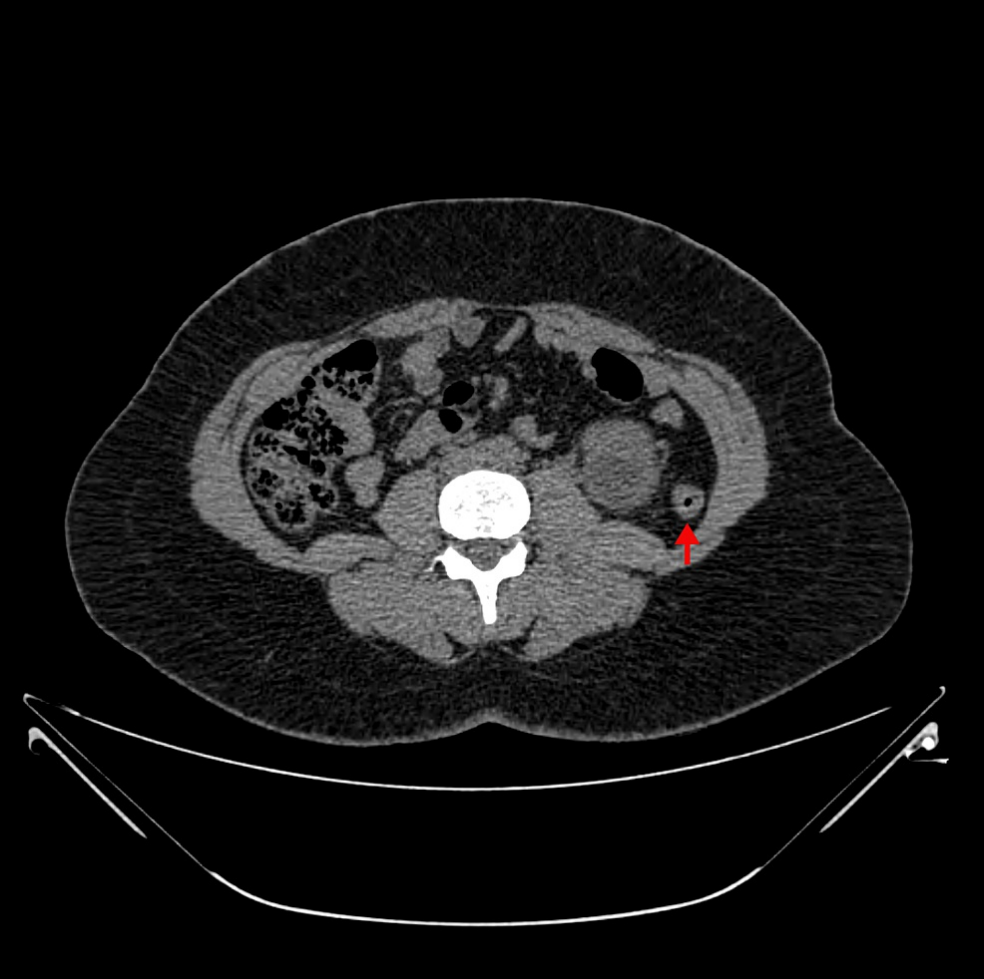
CT scan of abdomen - axial slice at lower pole showing left retro-renal colon with air in the lumen

A 38-year-old female presented to the Urology clinic with a history of recurrent bilateral flank pain and pyelonephritis for the past four years. She was known to have large bilateral renal pelvis stones with severe hydronephrosis. The right-sided stone was 2.0cm x 1.3cm and the left-sided stone was 2.5cm x 1.6cm. There was no Urology expertise available previously and the patient was financially unable to travel abroad for intervention. Her serum creatinine over the course remained normal and no nuclear medicine was available to further assess renal function. When endourology expertise became available a cystoscopy and bilateral JJ ureteric stent placement was attempted. This failed as no contrast or wire could get past the left-sided stone into the kidney. Contrast only, but no wire could get past the right-sided stone into the right kidney. Neither expertise nor equipment was available to perform a percutaneous nephrostomy. Equipment was unavailable to perform a ureteroscopy and lithotripsy. The patient was followed up with serial serum creatinines for another 12 months until equipment and expertise became available to perform a PCNL, however, on further image review for surgical planning, a left RRC was discovered (Figures 1-3). The decision was made instead for an open pyelolithotomy via the subcostal route to allow for adequate mobilization of the RRC.

**Figure 4:**
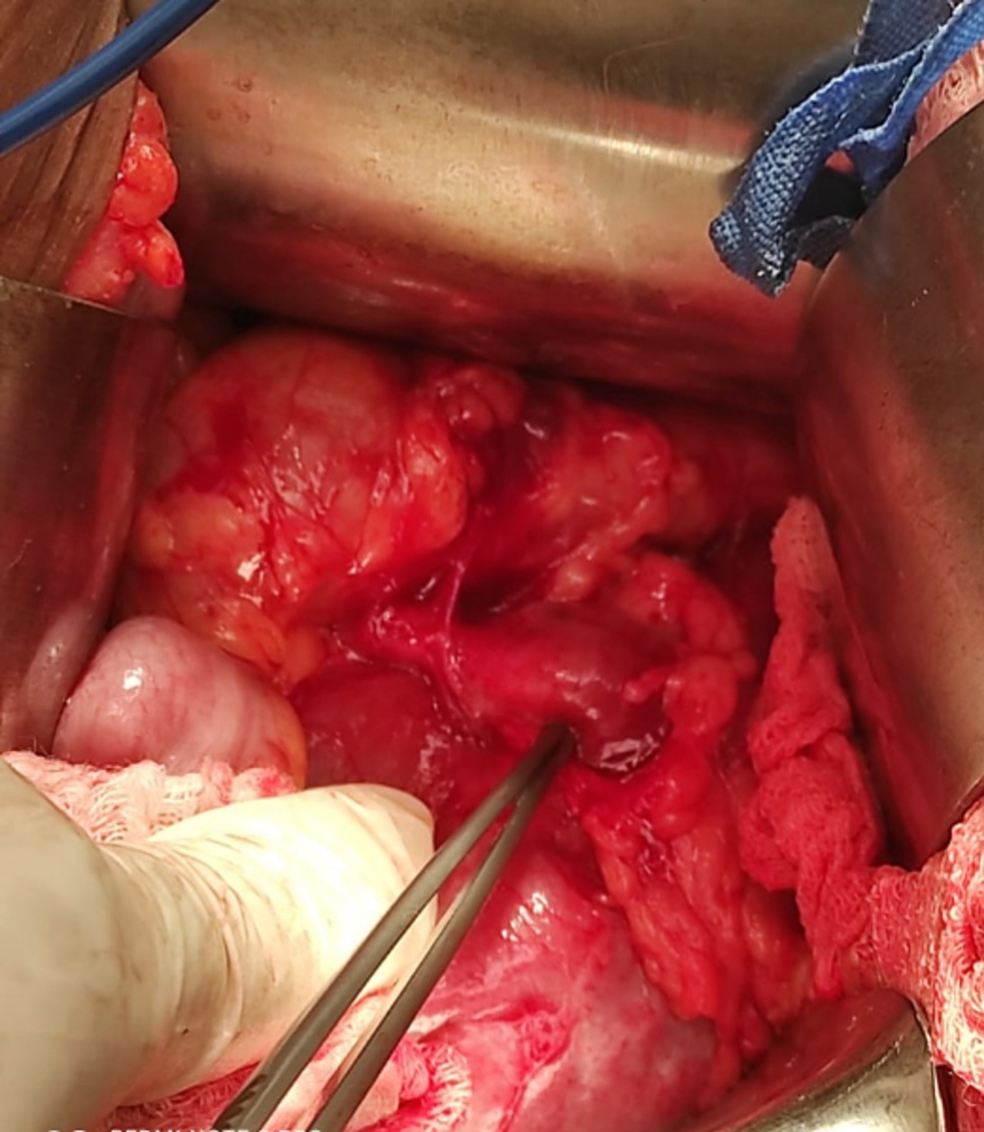
Intraop photo – left kidney with dilated renal pelvis, medialized colon

**Figure 5:**
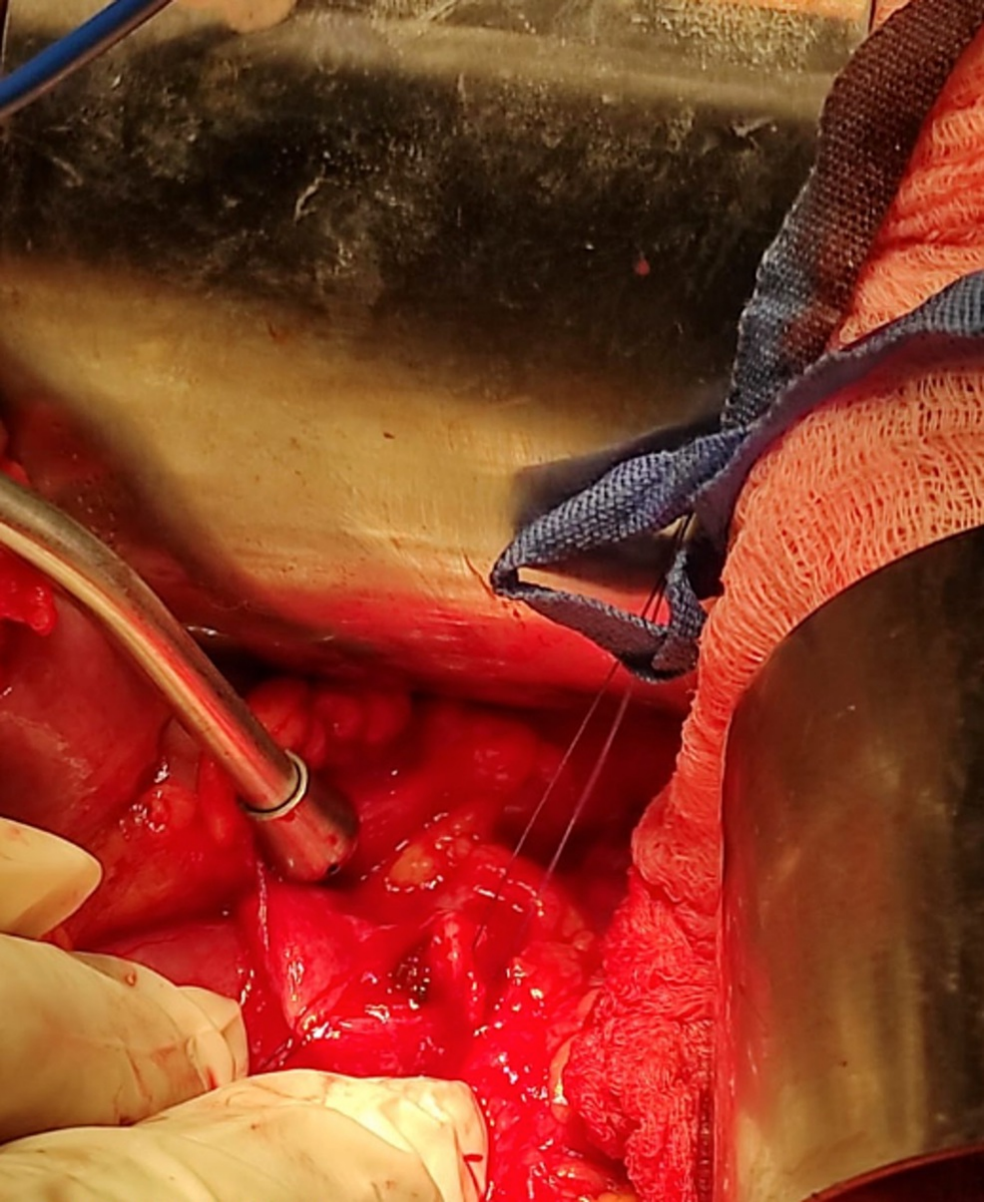
Intraop photo – opening renal pelvis between stay-sutures

**Figure 6:**
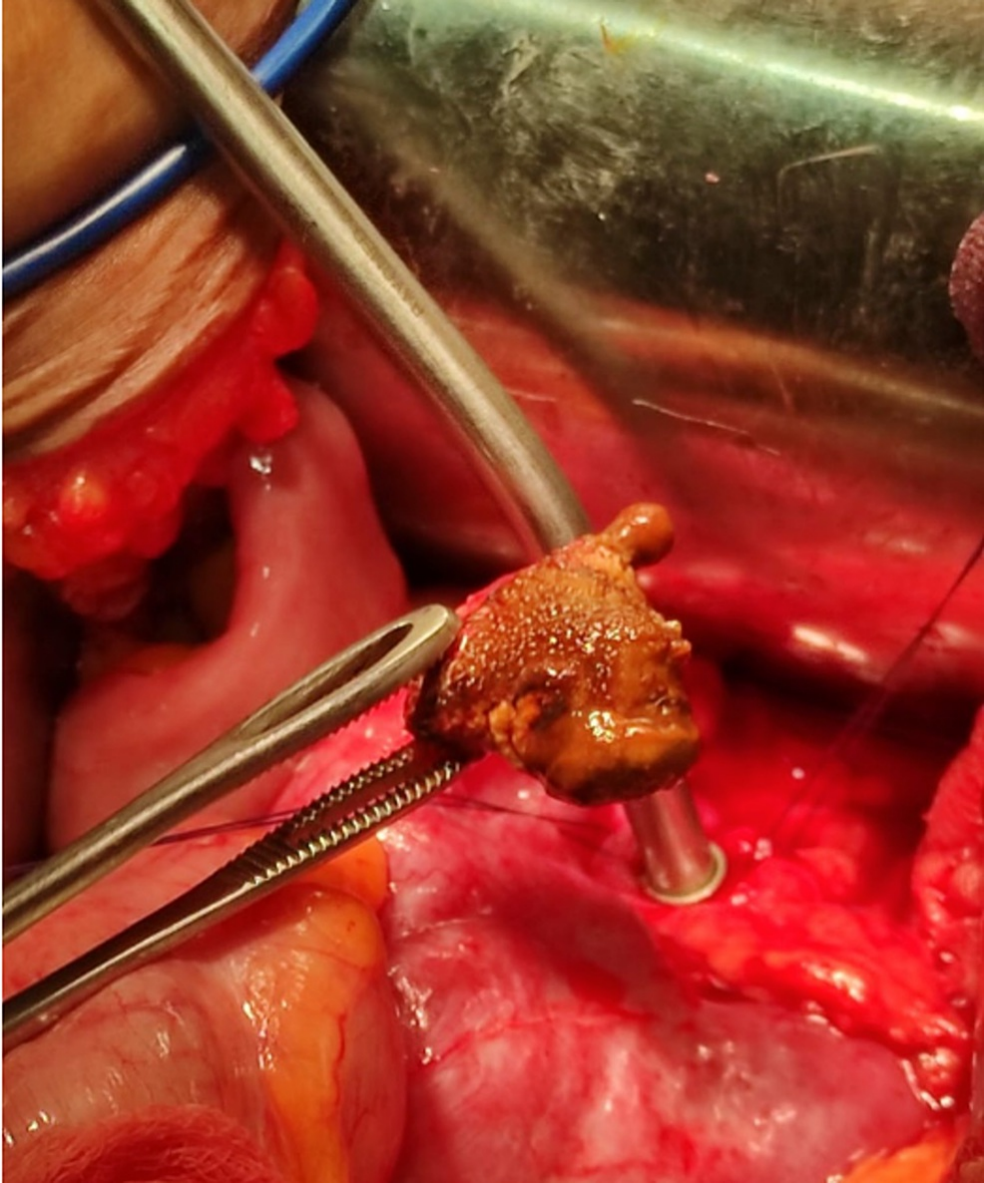
Intraop photo – stone extracted from the renal pelvis

**Figure 7:**
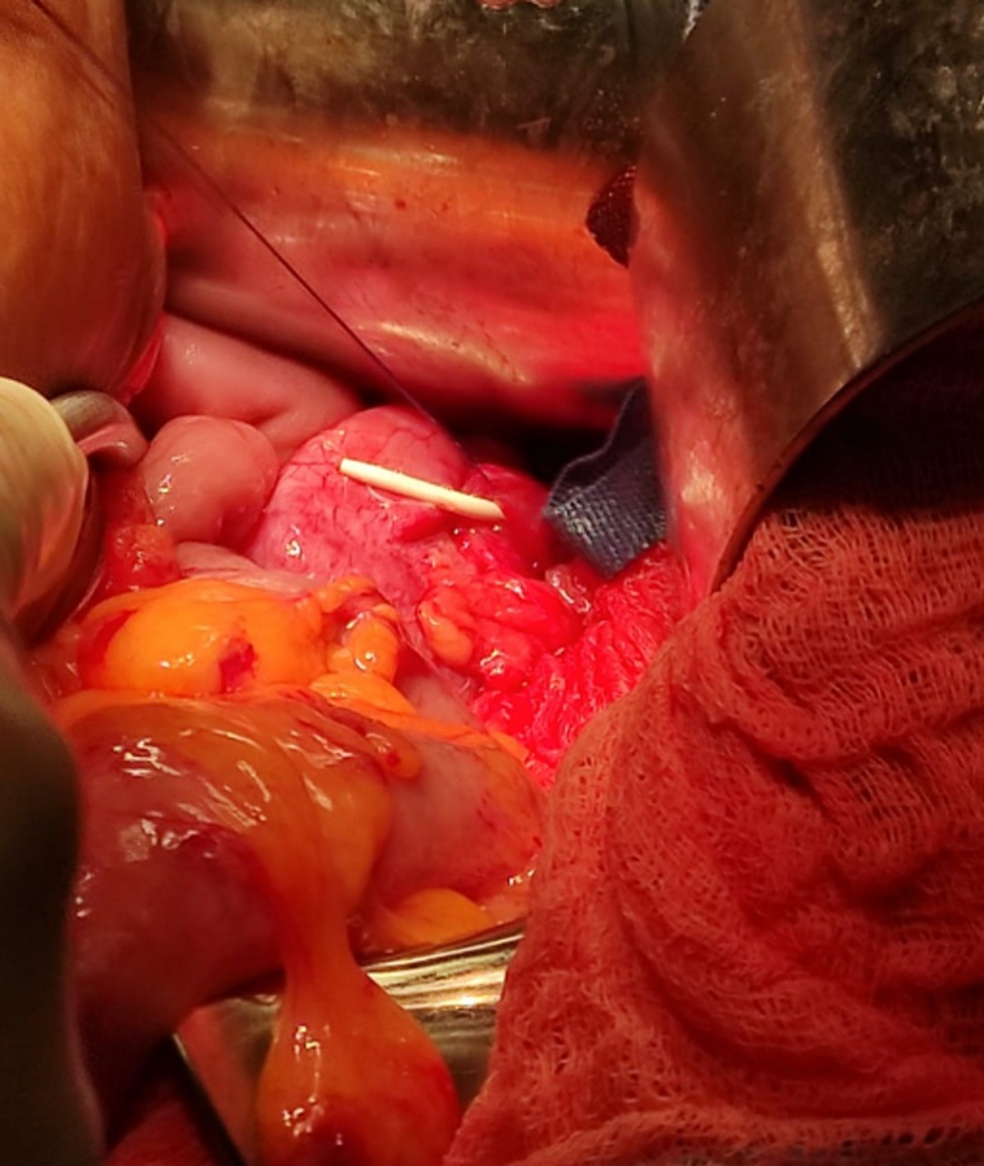
Intraop photo – renal pelvis closed over JJ ureteric stent

**Figure 8:**
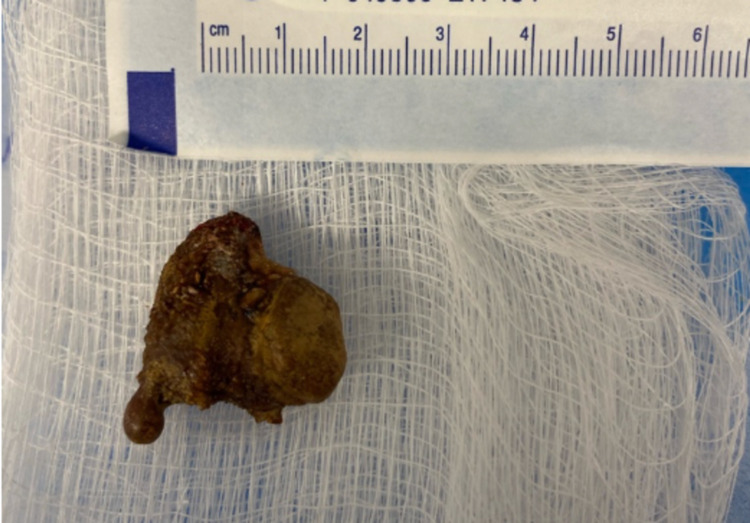
Intraop photo – renal stone

Under general anesthesia, a left subcostal incision to enter the abdominal cavity was made. The descending colon was mobilized medially to expose the renal hilum. A U-shaped incision was made in the renal pelvis between stay sutures. The stone was removed with a stone grasping forceps. The renal pelvis was close with 3-0 Vicryl sutures over a 6fr 22cm stent (Figures 4-8). This was accomplished in 110 mins. Blood loss was 200mls. The patient was discharged two days postoperatively with a plan for stent removal in six weeks and for surgical planning for the right-sided stone.

## Discussion

The European Association of Urology guidelines of nephrolithiasis recommend PCNL as the first choice for symptomatic stones at >20mm. They also do acknowledge the role of open or laparoscopic surgery in situations where percutaneous means are unlikely to be successful or if multiple endoscopic approaches have failed [1]. As technology and expertise improve for Urologists, the role of open stone surgery continues to diminish.

Very little is now published about open stone surgery. In 2012, El-Hussainy and Buccholz expounded on the diminishing indications but highlighted the relevance of these techniques in developing countries [2]. Data from the National Inpatient Sample database from 2001-2014 in the USA revealed that open stone surgery was performed on 2.6% of patients [3]. In this case, an RRC was discovered preoperatively on image review for surgical planning and this influenced the decision for an open pyelolithotomy instead of a PCNL.

Since the early 1980s, the anatomical relationship between the colon and the kidney has been studied [4,5,6]. The significance of this relationship became more prominent with the rise of percutaneous renal access. It was LeRoy et al. who used the term “retro-renal colon” to describe the position of the colon in two cases where percutaneous renal access was performed prior to nephrolithotomy. It was during a nephrolithotomy that it was discovered that the tract had gone through the colon in both cases [7].

Since then, many authors have tried to characterize the incidence of the RRC. Sherman et al. in their study of 394 patients found it present in (27) 9.9% of patients. It was found more commonly on the left (18) 4.6% vs right (4)1% and it was bilateral in (5) 1.3% of the patients they studied [8]. Boon et al. found an even higher incidence in their cohort of 333 patients (16%). They also used a more practical definition, the “line of the percutaneous tract” (through the postero-lateral edge of the vertebral body through the middle of the renal hilum) [9]. This line was also described by Prassopoullos et al. and these authors noted the RRC to be more common at the lower pole and in females [10]. Hadar and Gadoth found a higher incidence in females as well and this was postulated to be as a result of the near absence of perinephric fat in females [11].

Cross-sectional imaging with CT scanning is mostly routine in modern endourology however percutaneous renal access is sometimes performed without this. In the series by Balasar et al., the two cases of colonic perforation occurred in the cases where CT scanning was not performed [8]. A keen review of the position of the colon in relation to the kidney and area of interest is necessary. The method using the line described by Prassopoullos et al. is reproducible and accurate [10]. The method by Hadar and Gadoth using quadrants is also helpful in planning the surgical approach [11].

On detection of a retro-renal colon, a number of options exist for the safe removal of renal stones. Zgheib et al. demonstrated that percutaneous access can be safely achieved by using CT guidance [12]. In the index case, Interventional Radiology expertise and equipment were not available. Ureteroscopy and lithotripsy would have also been an option given the availability of the equipment. Given the size of the stone, it may have required multiple treatments. In this resource-limited setting, we have demonstrated that an open pyelolithotomy is a safe alternative to remove the stone and prevent colonic injury. It stands to reason, where the resources and expertise is available, the pyelolithotomy can be performed either laparoscopically or robotically to avoid injury to the RRC.

Failure to recognize an RRC can result in colonic perforation. Colonic perforation complicates 0.3% of PCNL procedures [13]. A series by Reddy and Shaik also demonstrated that although rare, only two cases of colonic perforations occurred in patients where preoperative CT scans were not done [14].

Colonic injury with percutaneous renal access can result in sepsis, peritonitis, abscess formation, or fistula formation [14]. Early detection and conservative or endoscopic management with a course of antibiotics has been reported to have good success rates. Traxer described that staged withdrawal of the nephrostomy tube into the colon to control bowel effluent, and then into the pericolic space was an important step for management. This plus the placement of a ureteric stent resulted in complete resolution in most patients. Failed resolution or intraperitoneal perforation, however, may require open intervention [15].

## Conclusions

Open stone surgery is rarely performed in this modern era of endourology. Percutaneous approaches are the standard of care for large renal stones. An RRC is a rare anatomical variant that may increase the risk of colonic injury during PCNL. A CT scan is essential pre-operatively to identify an RRC and plan the surgical approach to avoid colonic injury.

CT-guided access is a safe alternative if equipment and expertise are available. Another option making use of endourology expertise is ureteroscopy and lithotripsy. In the resource-limited setting, an open pyelolithotomy is still a safe and effective approach to remove renal stones when an RRC exists.
